# How to reduce learning curve in tricuspid valve surgery, the low-fidelity simulator as a choice for training in conventional and minimal invasive surgery

**DOI:** 10.1186/1749-8090-8-S1-P140

**Published:** 2013-09-11

**Authors:** A Hossien, I Khan, H Subhani, S Ashraf

**Affiliations:** 1 Cardiothoracic Surgery Department, Morriston Hospital, Swansea, UK

## Background

Presently the training in tricuspid valve (TV) surgery is difficult due to limited exposure of the TV, 3D complexity of TV components and repair procedures. Therefore we propose a portable simulator for cardiac trainees and junior surgeons to be proficient in TV surgical techniques and to minimize the learning curve. The simulator can be used for an unrestricted number of procedures. It is made with the lowest possible fidelity focusing on availability and cost-containment of the exercise.

## Methods

The TV simulator was made from a sponge that can be placed inside of any box or on any board. The simulator can be equipped with a drain pipe for minimal invasive purposes. The rings and valves were made from available materials. The sponge was covered with a surgical tape for effective manipulation.

## Results

The self-construction of the TV simulator results in improvement of the understanding of 3D TV anatomy. The usage of the sponge results in effective creation of TV components with similar properties to TV tissue. The usage of the simulator results in performing of all surgical procedures including TV ring annuloplasty, De Vega plasty, bicuspidization of the tricuspid valve, Edge to Edge, neo artificial chordae, pericardial augmentation of the leaflets and TV replacement as well. Unrestricted number of procedures can be performed after covering the sponge with surgical tape. Flexibility and cavity of the drain pipe allow the trainees to use the simulator for minimal invasive also.

## Conclusions

The surgical skills in TV surgery can be improved by usage of low fidelity simulator in classic and minimal invasive techniques. The high cost of the training residents and junior surgeons in TV surgery can be reduced effectively through the use of this low cost simulator and its accessories (bands, rings and valves etc.). The familiarity in performing surgical procedures results in reducing time consumed in the operation room and reduction of the learning curve.

